# Efficacy, safety and economy of denosumab and zoledronic acid in the treatment of bone metastases of solid tumors and multiple myeloma: a systematic review and meta-analysis

**DOI:** 10.3389/fonc.2025.1747354

**Published:** 2026-01-06

**Authors:** Li Zhong, Wei Chen, Da Zheng, Yuening Cao, Ling Zhu, Zefeng Zhu, Luqin Liao, Lilin Dai, Xiaotao Wang, Zheng Zeng

**Affiliations:** 1 Department of Pharmacy, The First Affiliated Hospital of Guilin Medical University, Guilin, China; 2 Department of Hematology, The First Affiliated Hospital of Guilin Medical University, Guilin, China

**Keywords:** bone metastases, denosumab, meta-analysis, skeletal-related events, systematic review, zoledronic acid

## Abstract

**Objective:**

To conduct a comprehensive comparison of the efficacy, safety, and cost-effectiveness of denosumab versus zoledronic acid in patients with bone metastases from solid tumors and multiple myeloma.

**Methods:**

A systematic search of PubMed, Web of Science, Embase, and major Chinese databases was performed for studies published up to 30 September 2025. Eligible evidence included randomized controlled trials, cohort studies, and pharmacoeconomic analyses. Random-effects models were applied for quantitative synthesis. The certainty of evidence for key outcomes was assessed using the GRADE framework.

**Results:**

Twenty-one studies were included. Moderate-certainty evidence indicates that denosumab likely delays the time to first skeletal-related event (SRE) (HR = 0.85, 95% CI: 0.79–0.93) and time to first and subsequent SREs (HR = 0.86, 95% CI: 0.76–0.97) relative to zoledronic acid. Subgroup analyses demonstrated that this benefit is pronounced in solid tumors but not observed in multiple myeloma. For survival outcomes, moderate-certainty evidence suggests little to no difference in overall survival (HR = 0.97, P = 0.49) or progression-free survival (HR = 0.99, P = 0.86). Low-certainty evidence suggests that denosumab may reduce the risk of any adverse events (OR = 0.70, P = 0.04) and nephrotoxicity (OR = 0.65, P = 0.02). Pharmacoeconomic evaluations revealed marked geographic heterogeneity: denosumab was generally cost-effective in high-income settings with higher willingness-to-pay thresholds, whereas in resource-limited regions, zoledronic acid remained the more economically favorable option.

**Conclusion:**

Denosumab probably confers superior protection against SREs in patients with solid tumors and demonstrates a potentially improved renal safety profile compared with zoledronic acid. However, its cost-effectiveness varies substantially across healthcare systems and is strongly shaped by regional pricing structures and willingness-to-pay thresholds. Clinical adoption should therefore consider tumor biology, safety characteristics, and local economic capacity.

**Systematic review registration:**

https://www.crd.york.ac.uk/prospero/, identifier CRD420251020691.

## 1 Introduction

Bone metastasis is one of the most prevalent and debilitating complications of advanced malignancies. It is estimated that 70–80% of patients with breast or prostate cancer and 30–40% of those with lung cancer develop bone metastases during the course of their illness (1–3). Nearly all patients with multiple myeloma (MM) experience osteolytic lesions of varying severity (4). The development of bone metastases is a complex pathological process in which tumor cells promote osteoclast overactivation through the secretion of multiple cytokines (such as PTHrP, IL-6, and TNF-α), thereby disrupting bone homeostasis and resulting in osteolytic, osteoblastic, or mixed bone lesions (5, 6). This “vicious cycle” not only facilitates tumor progression and dissemination within bone tissue but also increases skeletal fragility and further perturbs bone remodeling. Clinically, it ultimately manifests as a cascade of skeletal-related events (SREs), including pathological fractures, spinal cord compression, and the need for radiotherapy or surgical intervention to treat bone pain or osteolytic lesions (7, 8). SREs substantially increase patient morbidity, leading to reduced mobility, impaired functional status, and diminished quality of life, and they are associated with increased analgesic use and higher hospitalization rates. Moreover, SREs adversely affect prognosis and impose a considerable burden on healthcare resources, posing a major challenge for clinical management. Therefore, delaying or preventing the occurrence of SREs remains a key therapeutic goal in the comprehensive management of bone metastases in patients with solid tumors and MM.

At present, the main pharmacological agents used in clinical practice to prevent SREs are bisphosphonates and receptor activator of nuclear factor kappa-B ligand (RANKL) inhibitors (9). Zoledronic acid (ZA), a first-line bisphosphonate, ameliorates bone metabolic imbalance by inhibiting osteoclast activity and is widely used for the treatment of bone metastases from various solid tumors and MM (10, 11). Although its efficacy is well established, the risk of nephrotoxicity, ZA-associated acute-phase reactions, and its intravenous route of administration limit its broader use. In contrast, denosumab (Dmab), the first fully human monoclonal antibody targeting RANKL, inhibits osteoclast formation and activity by blocking the RANKL–RANK signaling pathway (12). Clinical studies have shown that, in patients with bone metastases (BMs) and MM, Dmab significantly delays the time to first SRE, reduces the risk of subsequent SREs, and is superior to ZA in delaying pain progression and reducing nephrotoxicity (13, 14). In addition, Dmab is administered subcutaneously, does not require routine monitoring of renal function, and is associated with higher patient adherence. However, Dmab may confer a relatively higher risk of hypocalcemia. Furthermore, drug acquisition costs and the associated long-term economic burden remain important considerations in clinical decision-making.

Despite these clinical findings, inconsistencies remain regarding the relative efficacy, safety, and pharmacoeconomic profiles of Dmab and ZA across different tumor types. Against this background, we conducted a systematic review and meta-analysis to comprehensively compare the efficacy, safety, and cost-effectiveness of Dmab versus ZA in patients with BMs and MM, with the aim of providing robust evidence to inform clinical decision-making and health policy development.

## 2 Materials and methods

### 2.1 Literature search strategy

This systematic review and meta-analysis were conducted in accordance with the PRISMA 2020 guidelines (15) and were prospectively registered in PROSPERO (registration number CRD420251020691). A comprehensive literature search was performed in Web of Science, the Cochrane Library, PubMed, Embase, Wanfang Data, CNKI, and the Chinese Biomedical Literature Database from database inception to September 30, 2025. The search strategy combined Medical Subject Headings (MeSH) and free-text terms related to “denosumab”, “zoledronic acid”, “bone metastasis”, and “multiple myeloma”, among others.

### 2.2 Inclusion and exclusion criteria

Inclusion criteria: Studies were included if they met all of the following conditions: (1) Participants: patients diagnosed with bone metastases from solid malignancies or with bone lesions secondary to MM; (2) Interventions: the experimental group received Dmab, whereas the control group received ZA, with no restrictions on dosing regimen or duration of treatment; (3) Outcomes: studies were required to report at least one of the following: effectiveness outcomes, including incidence of SREs, time to first SRE, overall survival, and progression-free survival; safety outcomes, including incidence of adverse events, incidence of severe adverse events (grade ≥3), incidence of common adverse events (such as fever, nausea, nephrotoxicity, fatigue, and anemia), and incidence of drug-related osteonecrosis of the jaw; and economic outcomes, including quality-adjusted life-years (QALYs), incremental cost, and incremental cost-effectiveness ratio (ICER); (4) Study design: randomized controlled trials and retrospective studies; and (5) Language: published in English or Chinese.

Exclusion criteria: Studies were excluded if they met any of the following conditions: (1) duplicate publications; (2) studies with missing key outcome data, such as efficacy or safety outcomes; (3) studies from which key outcome indicators could not be reliably extracted; (4) reviews, case reports, animal or *in vitro* studies, conference abstracts, letters, dissertations, and other non–peer-reviewed literature; (5) studies for which full-text articles were unavailable; (6) studies judged to be of low methodological quality; and (7) publications in languages other than English or Chinese.

### 2.3 Literature screening and data extraction

Two reviewers independently performed study selection and quality assessment. EndNote X9 software was used to manage the search results, and duplicate records were removed using both automated and manual procedures. Titles and abstracts were screened to exclude studies that clearly did not meet the predefined inclusion criteria. Subsequently, the full texts of potentially eligible articles were reviewed in detail to identify studies that fulfilled all eligibility criteria. Any disagreements between the two reviewers were resolved by discussion or, if necessary, in consultation with a third reviewer. To ensure methodological rigor, inter-rater reliability was evaluated using Cohen’s kappa statistic. Data from the included studies were organized in Excel 2019, and a standardized data extraction form was used to collect information from each eligible study. Extracted variables included article title, first author, year of publication, study design, sample size, details of the intervention and control, outcome measures, and other relevant characteristics. Every effort was made to ensure data completeness; when important data were missing, the corresponding authors were contacted to obtain the required information (**Supplementary Table S3**). After data extraction, all entries were cross-checked for accuracy, and any discrepancies were resolved by consensus between the two reviewers or, when necessary, through consultation with a third reviewer.

### 2.4 Literature quality evaluation

The Cochrane risk-of-bias assessment tool (16) was used to evaluate the risk of bias in the included randomized controlled trials. This tool includes seven domains: random sequence generation, allocation concealment, blinding of participants and personnel, blinding of outcome assessment, incomplete outcome data, selective reporting, and other sources of bias. Each domain was judged as having “low risk”, “high risk”, or “unclear risk” of bias. For cohort and case-control studies, the Newcastle–Ottawa Scale (NOS) for non-randomized studies (17) was used to assess quality across three domains: selection of study participants, comparability between groups, and assessment of outcomes. Risk of bias was judged using the semi-quantitative “star” rating system. In this system, the comparability domain can receive up to two stars, whereas each of the remaining items can receive at most one star. The maximum total score is 9 stars, with higher scores indicating higher methodological quality. Studies with scores of 0–3 were classified as low quality, those with scores of 4–6 as moderate quality, and those with scores of 7–9 as high quality.

The CHEERS 2022 checklist (18) was used to evaluate the reporting quality of pharmacoeconomic studies. This checklist consists of 28 items. For each item, full compliance was scored as 1 point, partial compliance as 0.5 points, and non-compliance or non-applicability as 0 points. Total scores ≥23.8 were considered excellent, 19.6 to <23.8 good, 15.4 to <19.6 acceptable, and <15.4 unacceptable.

### 2.5 Statistical analysis

Data analysis was conducted using Review Manager (RevMan) version 5.4. For time-to-event outcomes, hazard ratios (HRs) with 95% confidence intervals (CIs) were used as the summary effect measure. For dichotomous outcomes, odds ratios (ORs) with 95% CIs were calculated, and for continuous outcomes, mean differences (MDs) with 95% CIs were reported. Heterogeneity across studies was assessed using both the P-value (with P < 0.10 indicating statistical heterogeneity) and the I^2^ statistic. According to established criteria, I^2^ values of 0–40% represent low heterogeneity, 30–60% moderate heterogeneity, and 50–90% substantial heterogeneity.

Given the clinical and conceptual heterogeneity inherent across tumor types, study designs, baseline disease characteristics, and outcome definitions, we applied random-effects models throughout all meta-analyses, regardless of the magnitude of statistical heterogeneity. This approach ensures methodological conservatism and provides more reliable pooled estimates.

To explore potential sources of heterogeneity, pre-specified subgroup analyses were conducted based on primary tumor type (breast cancer, prostate cancer, multiple myeloma, and other solid tumors).

To evaluate the robustness of the results, we additionally performed systematic leave-one-out sensitivity analyses for all major outcomes, in which each study was sequentially excluded and the pooled effect re-estimated. All statistical tests were two-sided, and a P-value < 0.05 was considered statistically significant.

### 2.6 GRADE evidence grading system

The quality of evidence was assessed using GRADE profiler version 3.6 according to the Grading of Recommendations Assessment, Development and Evaluation (GRADE) approach, and was classified into four levels: high, moderate, low, and very low. The assessment considered five domains: risk of bias, inconsistency, indirectness, imprecision, and publication bias.

## 3 Results

### 3.1 Study selection

**Figure 1:**
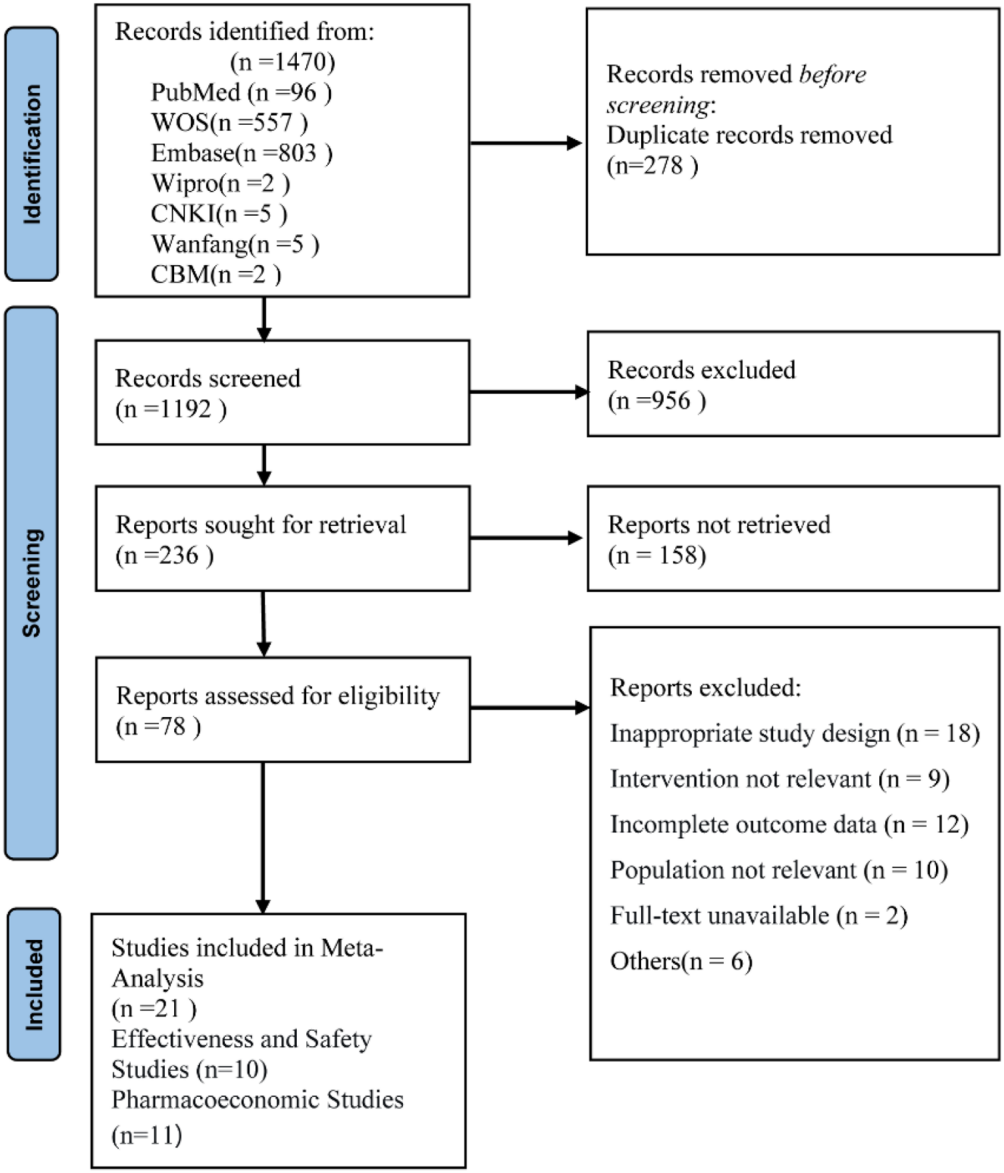
Literature screening flowchart.

A total of 1, 470 records were initially identified through the predefined search strategy. After duplicate removal using EndNote X9, 1, 193 unique records remained. Following title and abstract screening and subsequent full-text evaluation against the eligibility criteria, 21 studies were ultimately included in the review. Agreement between reviewers during the full-text screening stage was substantial, with a Cohen’s kappa of 0.75. The detailed study selection process is illustrated in **Figure 1**. The detailed search strategies for all databases are listed in **Supplementary Table S1**.

### 3.2 Basic characteristics of the included studies

A total of 21 studies were included in this review. Of these, 10 studies focused on clinical effectiveness and safety outcomes (14, 19–27), and the remaining 11 studies (28–38) addressed pharmacoeconomic evaluations. The basic characteristics of the included clinical studies and pharmacoeconomic studies are summarized in **Tables 1**, **2**, respectively.

**Table 1:** Fundamental characteristics of the included literature.

Study	Cancer types	Research type	Research duration (months)	Sample size	Age	Intervention measures	Outcome indicators
Stopeck, A.2010 (22)	Breast	RCT	42	ZA:1020 Dmab:1013	ZA:56 Dmab:57	ZA:120mg Dmab:4mg	①②③④⑤⑥
Fizazi.2011 (14)	Prostate	RCT	41	ZA:943 Dmab:945	ZA:71 Dmab:71	ZA:120mg Dmab:4mg	①②③④⑤⑥
Henry, D.H.2011 (19)	All, excluding breast and prostate	RCT	34	ZA:878 Dmab:878	ZA:60 Dmab:61	ZA:120mg Dmab:4mg	①②③④⑤⑥
Vadhan-Raj, S.2012 (23)	All, excluding breast and prostate	RCT	34	ZA:878 Dmab:878	ZA:60 Dmab:61	ZA:120mg Dmab:4mg	①②
Rajie.2018 (20)	Multiple myeloma	RCT	34	ZA:852 Dmab:850	ZA:63 Dmab:63	ZA:120mg Dmab:4mg	①②③④⑤⑥
He. 2022 (25)	Prostate	Retrospective cohort study	3	ZA:9 Dmab:9	ZA:71 Dmab:75	ZA:120mg Dmab:4mg	⑤
Zhang. 2023 (27)	Prostate	Retrospective cohort study	3	ZA:56 Dmab:48	ZA:63 Dmab:65	ZA:120mg Dmab:4mg	⑥
He. 2024 (24)	Breast	Retrospective cohort study	24	ZA:30 Dmab:30	ZA:45 Dmab:43	ZA:120mg Dmab:4mg	⑤
Huang. 2024 (26)	Breast	Retrospective cohort study	18.3	ZA:66 Dmab:66	ZA:53 Dmab:58	ZA:120mg Dmab:4mg	⑤⑥
Scafetta.2025 (21)	Breast	Retrospective cohort study	54.6	ZA:246 Dmab:618	ZA:61 Dmab:61	ZA:120mg Dmab:4mg	①

①Time to first SRE; ②Time to first and subsequent SREs; ③Overall Survival (OS); ④Progression-Free Survival(PFS); ⑤Overall incidence of Adverse Events; ⑥Incidence of Serious Adverse Events (SAEs).

**Table 2:** Basic characteristics of included pharmacoeconomic studies.

Study	Country	Research type	Study horizon	Perspective	Model	Interventions	Cancer	Mean costs (drug administration and SRE, $)			Incremental effect		ICER ($/QALY)	CHEERS (0-28)
								Dmab	ZA	Incremental cost($)	ΔQALYs	ΔSREs		
Xie, J.2011 (36)	USA	CEA	12months	Payer	Markov	Dmab VS ZA	Prostate	51, 012.19	39, 734.69	11, 277.50	NR	-0.20	564, 3804.04	26
			36months					10, 0272.07	6219.23	6131.76	NR	-0.28	3998, 295.95
Stopeck, A.2012 (32)	USA	CUA	Lifetime	US managed-care	Markov	Dmab VS ZA	Breast	150, 832.08	132, 139.62	18, 692.46	0.17	-0.99	109, 665.86	25.5
							CRPC	106, 290.35	96, 689.12	9601.23	0.14	-0.81	68, 656.68
							NSCLC	68, 188.36	62, 525.45	5662.91	0.06	-0.06	94, 401.72
Xie, J.2012 (35)	USA	CUA	12months	Payer	Markov	Dmab VS ZA	Breast	41, 777.23	32, 704.84	9072.39	NR	-0.06	NR	21.5
Lothgren, M.2013 (29)	Austria Sweden Switzerland	BIA	12months	Payer	Markov	Dmab VS ZA	Prostate	14, 038.35	16, 870.82	-2832.47	NR	-0.17	NR	20
							Breast	12, 916.67	15, 437.45	-2520.78		-0.15
							OST	17, 313.63	19, 917.11	-2603.48		-0.14
Snedecor, S. J.2013 (31)	USA	CUA	27 months	Payer	Markov	Dmab VS ZA	Breast	43, 393.78	33, 135.59	11317.92	0.007	-0.24	15, 28, 216.55	21.5
Cristino, J.2017 (28)	Czech Republic	CUA	Lifetime	Payer	Markov	Dmab VS ZA	Prostate	29, 583.92	27, 725.34	1858.58	0.09	-0.63	21, 365.03	20
							Breast	50, 087.89	48, 016.06	2071.83	0.09	-0.73	22804.18
							OST	26, 242.88	24, 834.79	1408.09	0.04	-0.27	33952.65
Raje, N.2018 (30)	USA	CUA	Lifetime	Payer	Markov	Dmab VS ZA	Multiple Myeloma	410, 284.74	347, 526.18	62, 758.56	0.24	-0.05	587, 333.47	26.5
Terpos, E.2019 (33)	Austria Belgium Greece Italy	CUA	Lifetime	Healthcare System	Markov	Dmab VS ZA	Multiple Myeloma	174, 864.93	137, 084.02	37, 780.91	0.21	-0.05	28, 800.12	25.5
Li We.2022 (38)	China	CUA	Lifetime	Whole society	Markov	Dmab VS ZA	Breast	38, 502.44	47, 324.23	-8821.79	0.11	-1.03	NR	21.5
							NSCLC	18, 621.30	21, 241.71	-2620.41	0.09	-0.34
							Prostate	29, 711.27	34, 859.22	-5147.95	0.05	-0.65
Wadhwa, R.2024 (34)	India	CUA	Lifetime	Healthcare System	Markov	Dmab VS ZA	Breast	12, 350.99	6219.23	6131.76	0.01	-0.28	485, 558.49	26
Chen.2025 (37)	China	CUA	Lifetime	Whole society	Markov	Dmab VS ZA	Multiple Myeloma	3040.05	1344.96	1695.09	0.06	NR	3568.70	22

CEA, cost-effectiveness analysis; CUA, cost-utility analysis; BIA, budget impact analysis; CRPC, castration-resistant prostate cancer; NSCLC, non-small cell lung cancer; OST, other solid tumors; NR, not reported. All costs are presented in USD. CHEERS score range: 0–28 points.

### 3.3 Quality assessment of the included studies

Among the ten included studies, five (14, 19, 20, 22, 23) were randomized controlled trials. For the domain of random sequence generation, four trials were judged to have a low risk of bias, whereas one study (19) was assessed as unclear risk due to insufficient information on the specific method used for randomization. Regarding allocation concealment, all five randomized studies clearly described appropriate procedures and were therefore rated as low risk.

**Figure 2:**
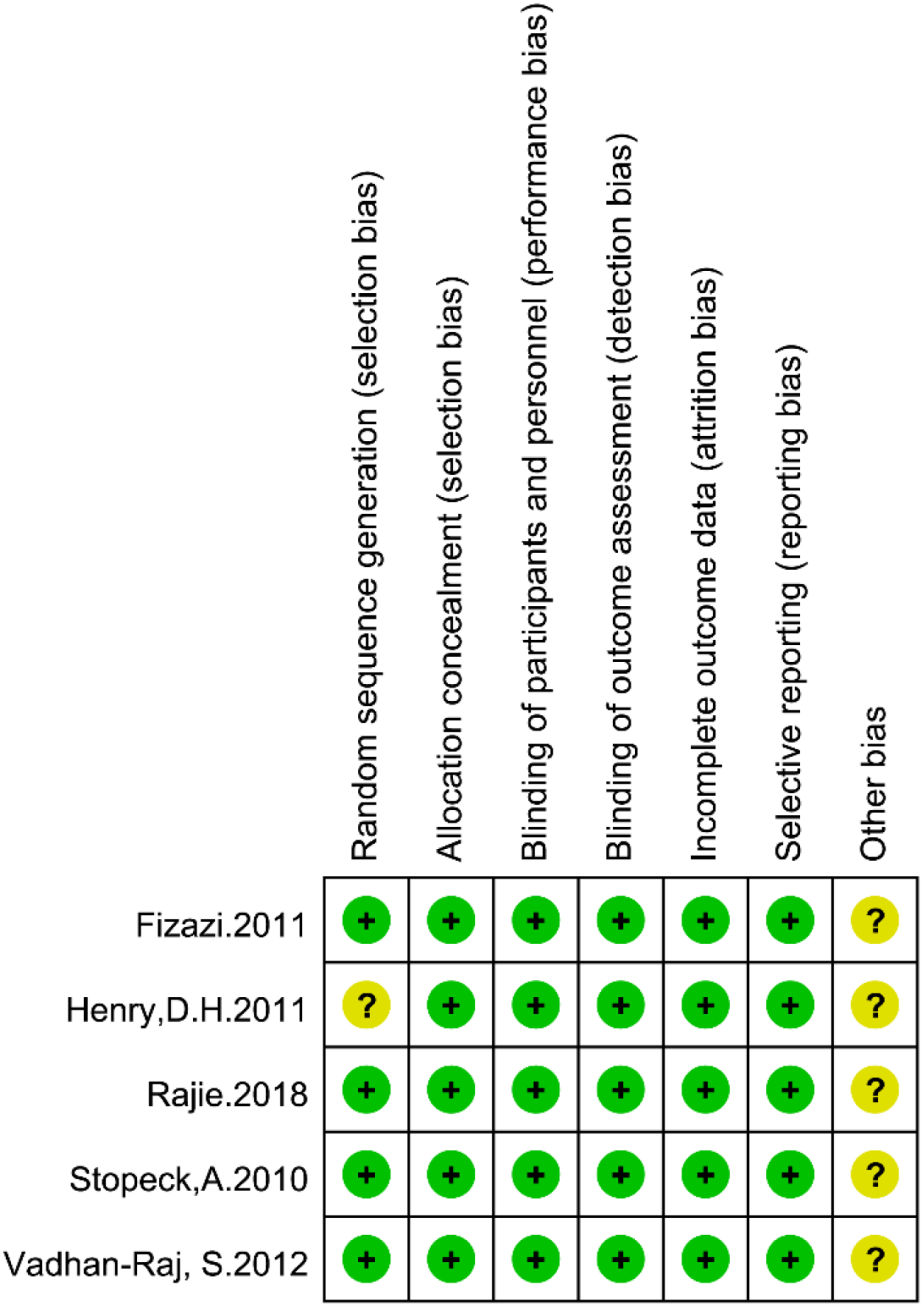
Quality evaluation of randomized controlled trial literature.

With respect to blinding, all five trials were considered to have a low risk of bias for both blinding of participants and personnel and blinding of outcome assessment. All studies reported complete outcome data, and no evidence of selective reporting was detected; therefore, both domains were assessed as low risk. For the domain of other potential sources of bias, all included studies were rated as having an unclear risk, primarily because the available information was insufficient to definitively rule out additional biases. The risk-of-bias summary and traffic-light plot were generated using Review Manager (RevMan) version 5.4, and the distribution of risk across individual studies is presented in **Figure 2**. The results of the quality assessment for the cohort studies are presented in **Supplementary Table S2**.

Among the eleven pharmacoeconomic studies (30, 32–34, 36) were of high quality, and six (28, 29, 31, 35, 37, 38) were of medium quality. Overall, most randomized controlled trials were judged to be at low to moderate risk of bias, and the majority of economic evaluations were of moderate to high quality.

### 3.4 Results of the meta-analysis

#### 3.4.1 Time to first SRE

**Figure 3:**
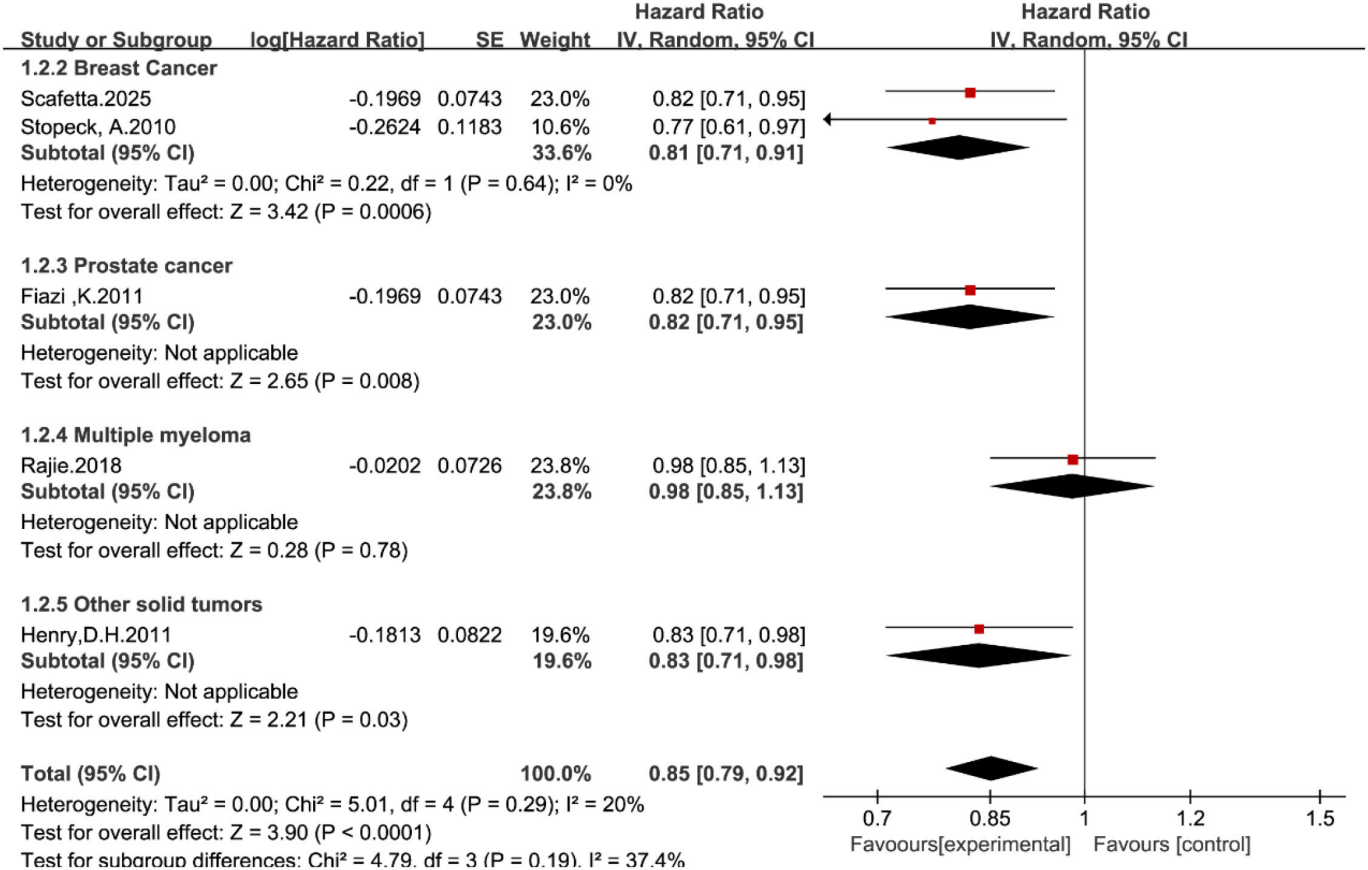
Forest plot of hazard ratios for time to first SRE by tumor type.

Five studies (14, 19–22) reported data on time to first SRE. In the overall pooled analysis using the random-effects model, denosumab significantly delayed the time to the first SRE compared with zoledronic acid (HR = 0.85, 95% CI: 0.79–0.93, P < 0.0001; **Figure 3**).

Subgroup analysis stratified by tumor type revealed distinct efficacy profiles across different malignancies. Denosumab likely reduces the risk of first SRE in the breast cancer subgroup (HR = 0.81, 95% CI: 0.71–0.91), the prostate cancer subgroup (HR = 0.82, 95% CI: 0.71–0.95), and the other solid tumors subgroup (HR = 0.84, 95% CI: 0.71–0.99).

In contrast, no significant benefit was observed in the multiple myeloma subgroup, where the hazard ratio was 0.98 (95% CI: 0.85–1.13, P = 0.78). This finding suggests that while denosumab appears superior to zoledronic acid in solid tumors with bone metastases, the two agents may have comparable efficacy in multiple myeloma. The test for subgroup differences showed moderate heterogeneity (I^2^ = 36.9%, P = 0.19), reflecting the variation in treatment response between solid tumors and myeloma.

#### 3.4.2 Time to first and subsequent SREs

**Figure 4:**
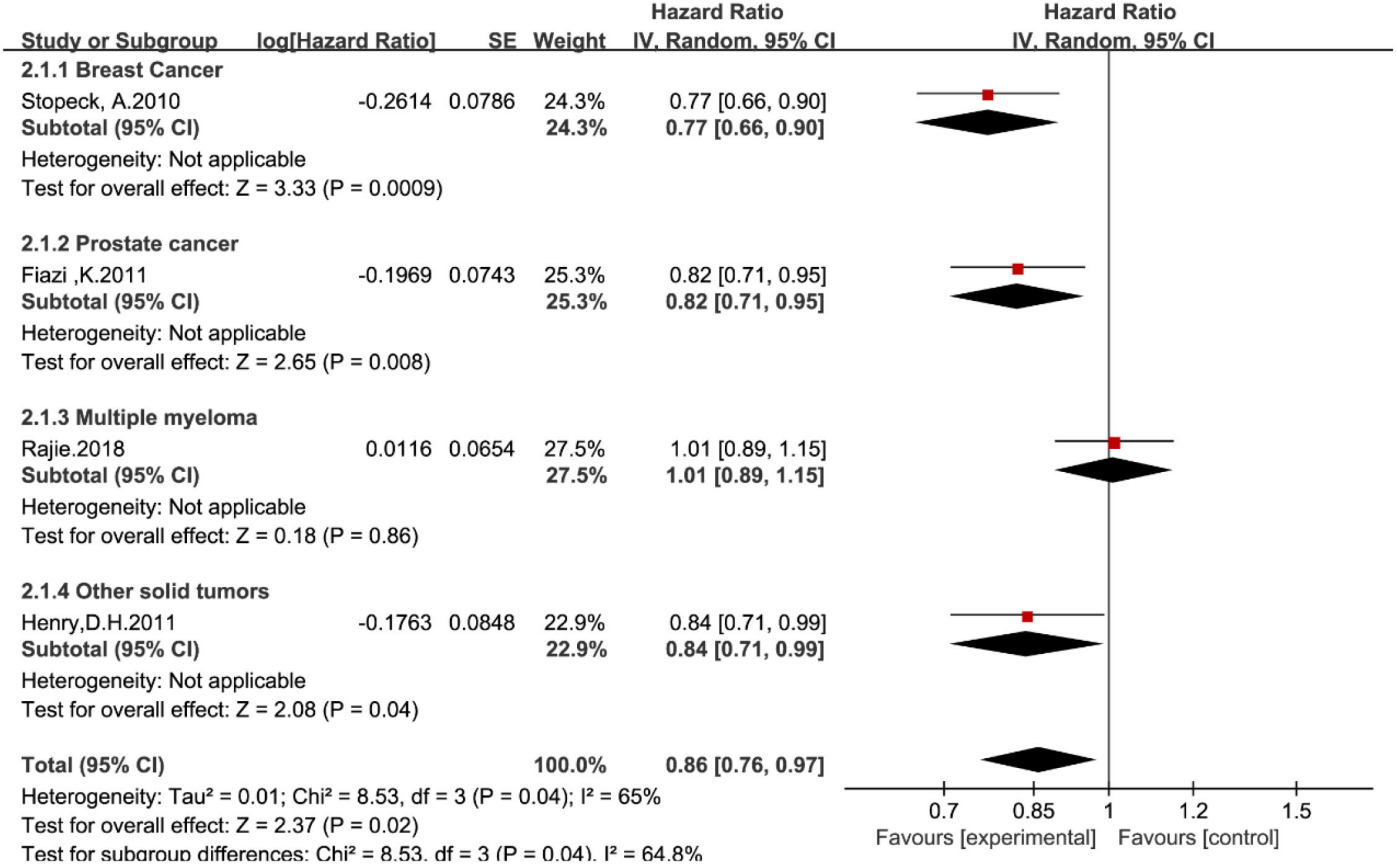
Forest plot of hazard ratios for time to first and subsequent SREs by tumor type.

Four studies (14, 19, 20, 22) reported the time to first and subsequent SREs. In the overall analysis, denosumab significantly reduced the risk of developing multiple SREs compared with zoledronic acid (HR = 0.86, 95% CI: 0.76–0.97; **Figure 4**).

However, subgroup analysis revealed significant heterogeneity driven by tumor type (P = 0.04). Denosumab demonstrated superior efficacy in breast cancer (HR = 0.77, 95% CI: 0.66–0.90) and prostate cancer (HR = 0.82, 95% CI: 0.71–0.95). In stark contrast, no benefit was observed in the multiple myeloma subgroup, with a hazard ratio of 1.01 (95% CI: 0.89–1.15). This statistically significant subgroup difference confirms that the therapeutic advantage of denosumab in preventing multiple skeletal events is specific to solid tumors and does not extend to multiple myeloma.

#### 3.4.3 Overall survival

**Figure 5:**
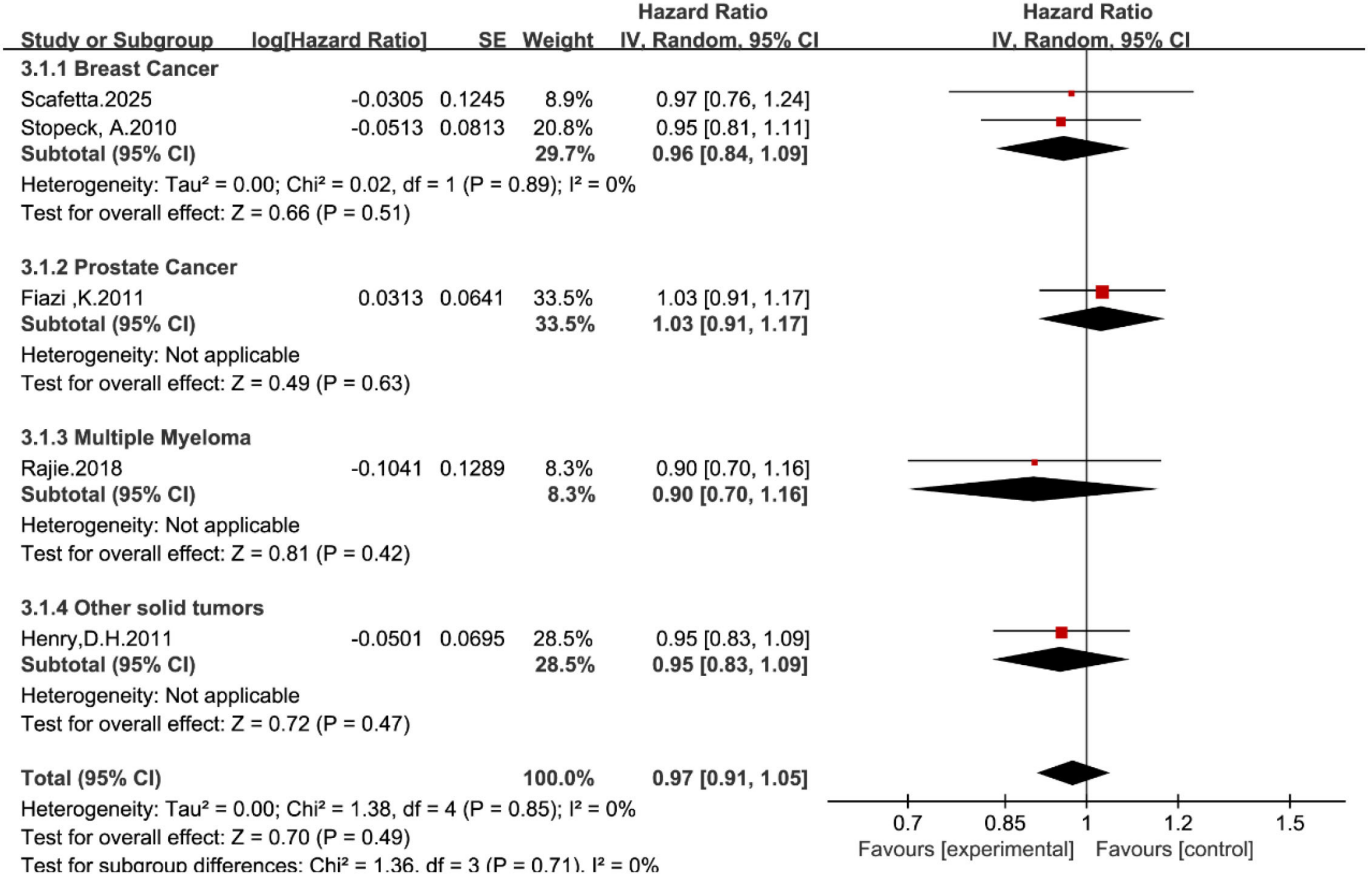
Forest plot of hazard ratios for overall survival by tumor type.

A total of five studies reported overall survival data (14, 19–22). In the pooled analysis using the random-effects model, no statistically significant difference was observed in overall survival between the denosumab and zoledronic acid groups (HR = 0.97, 95% CI: 0.91–1.05, P = 0.49; **Figure 5**).

Subgroup analysis stratified by tumor type confirmed the high consistency of this finding across all malignancies (P = 0.71). Denosumab did not confer a survival advantage compared with zoledronic acid in breast cancer (HR = 0.96, 95% CI: 0.84–1.09), prostate cancer (HR = 1.03, 95% CI: 0.91–1.17), multiple myeloma (HR = 0.90, 95% CI: 0.70–1.16), or other solid tumors (HR = 0.95, 95% CI: 0.83–1.09).

The complete absence of heterogeneity (I^2^ = 0%) further supports the conclusion that denosumab and zoledronic acid have comparable effects on patient survival, regardless of the primary tumor biology.

#### 3.4.4 Progression-free survival

**Figure 6:**
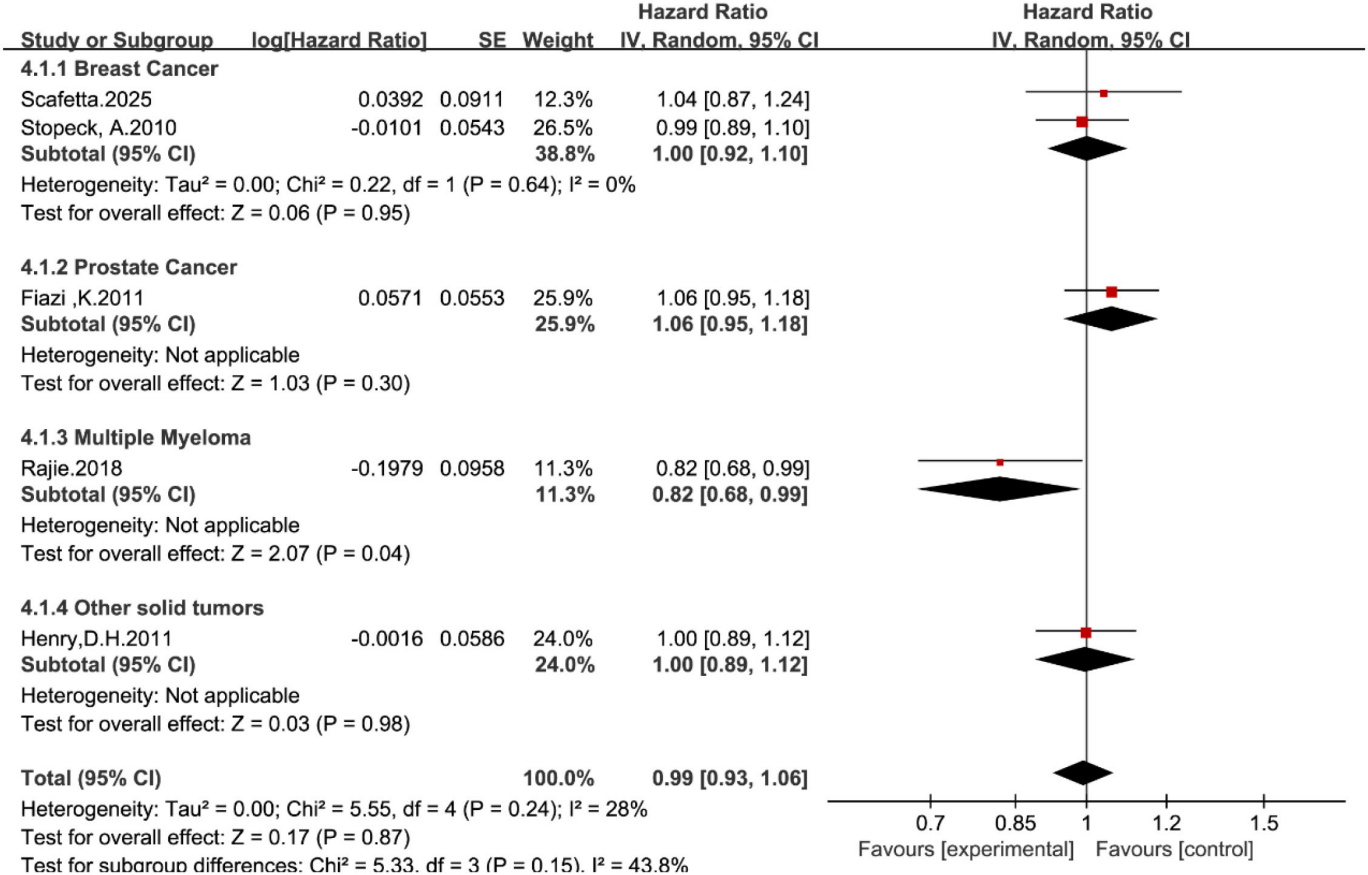
Forest plot of PFS hazard ratios stratified by tumor type.

Progression-free survival (PFS) data were available from five studies (14, 19–22). In the overall pooled analysis, no statistically significant difference was observed between denosumab and zoledronic acid (HR = 1.00, 95% CI: 0.93–1.07, P = 0.92; **Figure 6**). The overall heterogeneity was low (I^2^ = 28%).

Subgroup analysis stratified by tumor type revealed that for most solid tumors, the treatment effects were comparable. The hazard ratios for breast cancer (HR = 1.01, 95% CI: 0.92–1.12), prostate cancer (HR = 1.06, 95% CI: 0.95–1.18), and other solid tumors (HR = 1.00, 95% CI: 0.89–1.12) were all close to unity, indicating no difference in disease progression.

However, a notable exception was observed in the multiple myeloma subgroup, where denosumab was associated with a statistically significant improvement in PFS compared with zoledronic acid (HR = 0.82, 95% CI: 0.68–0.99, P = 0.04). Although the test for subgroup differences did not reach statistical significance (P = 0.15), this finding suggests a potential specific benefit in the myeloma population that contrasts with the results in solid tumors.

#### 3.4.5 Overall safety outcomes: any adverse events and serious adverse events

**Figure 7:**
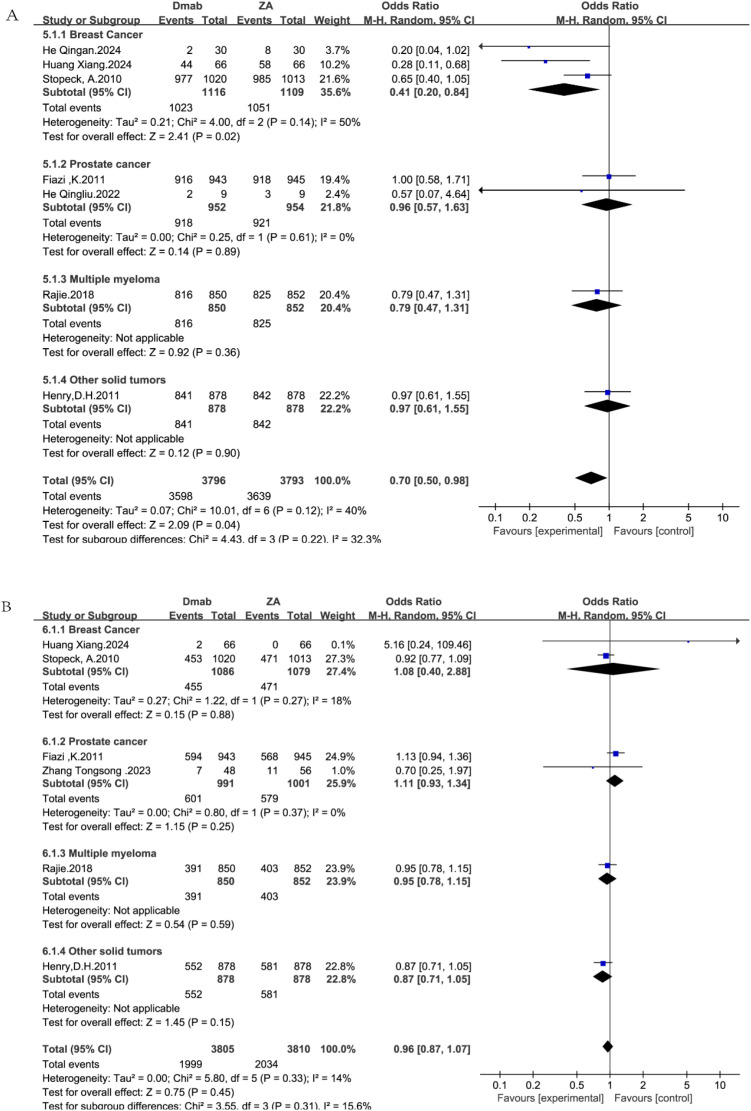
Forest plots for general safety outcomes: **(A)** any adverse events; **(B)** serious adverse events.

The general safety profile of denosumab versus zoledronic acid is summarized in **Figure 7**.

Seven studies (14, 19, 20, 22, 24–26) reported the overall incidence of adverse events (AEs). In the overall pooled analysis using the random-effects model, denosumab was associated with a statistically significant reduction in the risk of adverse events compared with zoledronic acid (OR = 0.70, 95% CI: 0.50–0.98, P = 0.04; **Figure 7A**).

Subgroup analysis stratified by tumor type showed variations in safety outcomes. A substantial reduction in adverse events was observed in the breast cancer subgroup (OR = 0.41, 95% CI: 0.20–0.84, P = 0.02). In contrast, the odds ratios for prostate cancer (OR = 0.96, 95% CI: 0.57–1.63), multiple myeloma (OR = 0.79, 95% CI: 0.47–1.31), and other solid tumors (OR = 0.97, 95% CI: 0.61–1.55) did not reach statistical significance, although point estimates generally favored denosumab.

The overall heterogeneity was moderate (I^2^ = 40%), and the test for subgroup differences was not statistically significant (P = 0.22), suggesting that while the magnitude of benefit may vary, the direction of the safety advantage is generally consistent.

Regarding the severity of adverse outcomes, six studies (14, 19, 20, 22, 26, 27) reported the incidence of SAEs. In the overall pooled analysis using the random-effects model, no statistically significant difference was observed between the denosumab and zoledronic acid groups (OR = 0.96, 95% CI: 0.87–1.07, P = 0.45; **Figure 7B**).

Subgroup analysis stratified by tumor type demonstrated consistent safety profiles across all malignancies (P = 0.31). The risk of serious adverse events was comparable between treatment arms in breast cancer (OR = 1.08, 95% CI: 0.40–2.88), prostate cancer (OR = 1.11, 95% CI: 0.93–1.34), multiple myeloma (OR = 0.95, 95% CI: 0.78–1.15), and other solid tumors (OR = 0.87, 95% CI: 0.71–1.05).

The low heterogeneity observed (I^2^ = 14%) reinforces the conclusion that the incidence of serious adverse events is similar for both agents, regardless of the primary tumor type.

#### 3.4.6 Nephrotoxicity and other specific adverse events

**Figure 8:**
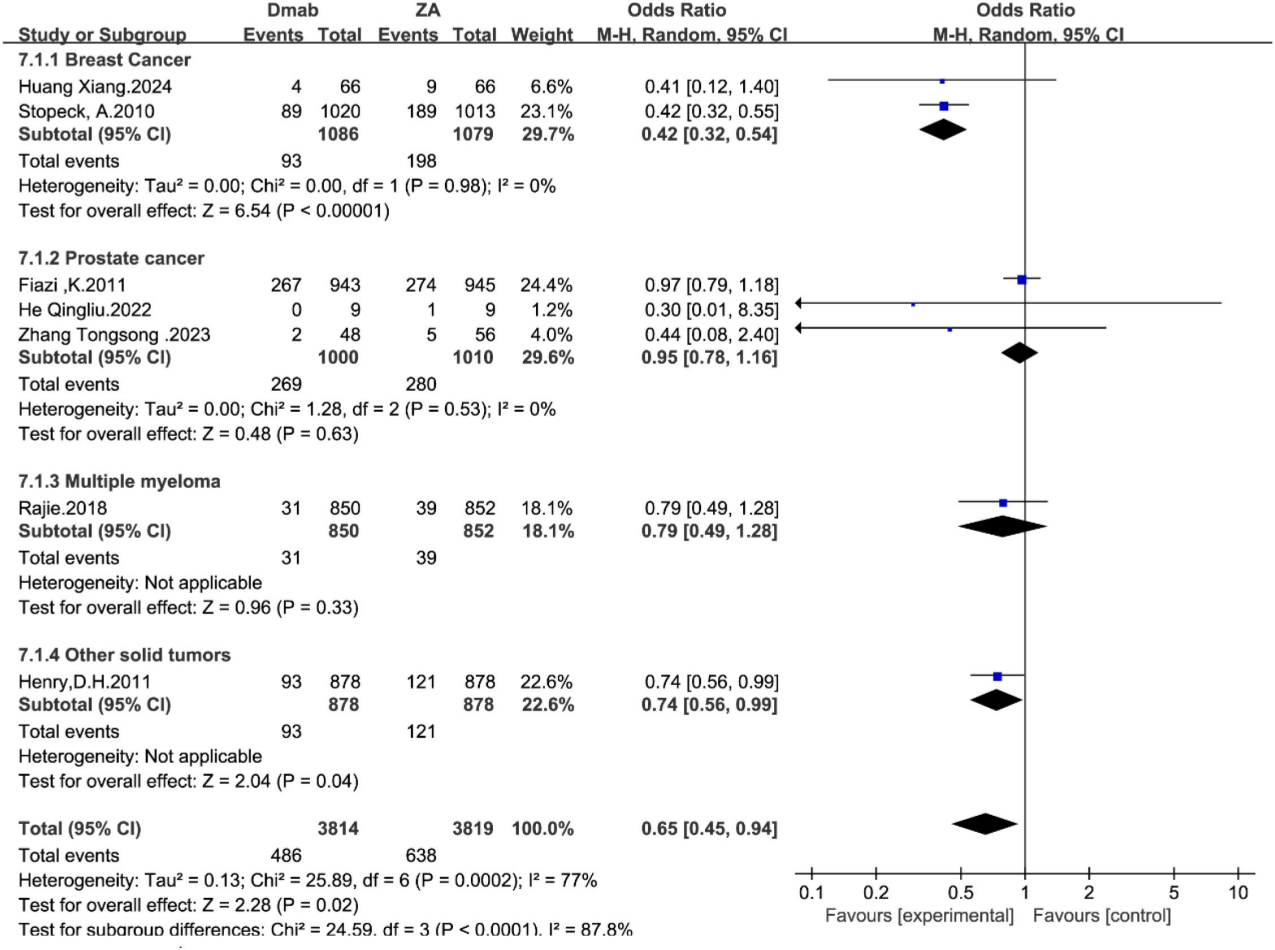
Forest plot of nephrotoxicity odds ratios stratified by tumor type.

Given the potential for nephrotoxicity associated with bisphosphonates, a dedicated subgroup analysis was performed for renal adverse events. In the pooled analysis using the random-effects model, denosumab demonstrated a significantly superior renal safety profile compared with zoledronic acid (OR = 0.65, 95% CI: 0.45–0.94, P = 0.02; **Figure 8**).

Subgroup analysis revealed that the magnitude of this benefit varied significantly across tumor types (P < 0.0001). The protective effect was most pronounced in the breast cancer subgroup (OR = 0.42, 95% CI: 0.32–0.54, P < 0.00001). While the point estimates for multiple myeloma (OR = 0.79) and other solid tumors (OR = 0.74) also favored denosumab, the difference did not reach statistical significance in these specific subgroups. In prostate cancer, the risk was comparable between the two arms (OR = 0.95).

Regarding other specific adverse events detailed in **Supplementary Table S4**, denosumab significantly reduced the risk of acute phase reactions (e.g., fever, flu-like symptoms) compared with zoledronic acid (P < 0.001). Conversely, the incidence of adverse events of special interest, such as osteonecrosis of the jaw (ONJ) and hypocalcemia, was comparable between the two treatment groups.

### 3.5 Sensitivity analysis

To evaluate the robustness of the findings, we conducted a systematic leave-one-out sensitivity analysis (**Supplementary Tables S5**-**S10**).

For the primary endpoint, time to first SRE, the results demonstrated high robustness. Sequential exclusion of individual studies yielded only minimal variation in the pooled hazard ratio (0.82–0.86), and statistical significance was maintained in all iterations (all P < 0.01). These findings indicate that the observed treatment benefit of denosumab is not driven by any single dominant trial.

For the composite endpoint of time to first and subsequent SREs, exclusion of the multiple myeloma study [Raje et al. (20)] eliminated statistical heterogeneity (I² decreased from 65% to 0%) and strengthened the estimated treatment effect. This pattern is consistent with the biological distinction between solid tumor bone metastases and plasma-cell–mediated bone disease. However, exclusion of other large pivotal trials [e.g., Stopeck et al. (22)] rendered the pooled effect non-significant (P > 0.05), suggesting that the statistical significance of this composite outcome is reliant on the robust statistical power contributed by these large-scale studies.

For any adverse events, the systematic leave-one-out analysis demonstrated that the pooled effect estimate remained stable across study exclusions. Notably, exclusion of the small-sample study [He et al. (25)] did not materially change the pooled odds ratio (OR = 0.70), indicating that this cohort did not meaningfully influence the overall estimate. However, the statistical significance of the safety benefit was sensitive to the aggregate sample size. Exclusion of larger trials [e.g., Stopeck et al. (22)] shifted the P-value to borderline significance (P > 0.05), although the direction of the effect consistently favored denosumab (OR < 1.0).

### 3.6 Assessment outcomes of the GRADE evidence grading system

The certainty of evidence for each outcome was systematically assessed using the GRADE framework, which evaluates five domains: risk of bias, inconsistency, indirectness, imprecision, and publication bias. Evidence for time to first SRE, time to first and subsequent SREs, OS, and PFS was rated as moderate certainty. Specifically, the certainty for time to first SRE, OS, and PFS was downgraded by one level due to serious risk of bias, primarily attributable to the inclusion of observational studies with inherent confounding risks. For the composite endpoint of time to first and subsequent SREs, certainty was downgraded by one level for serious inconsistency, reflecting substantial statistical heterogeneity (I² = 65%), largely driven by differential treatment effects in the multiple myeloma subpopulation. Evidence regarding the incidence of any adverse events and serious adverse events was rated as low certainty. This reflects a two-level downgrade due to very serious risk of bias, as a substantial proportion of data originated from observational studies susceptible to confounding and selection bias.

The complete GRADE evidence profile, detailing domain-specific assessments and the rationale for downgrading, is presented in **Supplementary Table S11** and summarized in **Table 3**, providing a transparent overview of both the effect estimates and the reliability of the supporting evidence.

**Table 3:** Summary of findings including GRADE certainty of evidence assessment.

Outcome	No. of studies	Effect estimate (95% CI)	I²	P value	Certainty of evidence (GRADE)
Time to first SRE	5	HR 0.85 [0.79, 0.93]	20%	0.0001	Moderate
Time to first and subsequent SREs	4	HR 0.86 [0.76, 0.97]	65%	0.02	Moderate
Overall survival	5	HR 0.97 [0.91, 1.05]	0%	0.49	Moderate
Progression-free survival	5	HR 0.99 [0.93, 1.06]	28%	0.86	Moderate
Any adverse events	7	OR 0.70 [0.50, 0.98]	40%	0.04	Low
Serious adverse events	6	OR 0.96 [0.87, 1.07]	14%	0.45	Low

### 3.7 Economic evaluation

A total of eleven pharmacoeconomic evaluations were identified, spanning diverse geographic settings including the United States, multiple European countries, and Asian regions such as China and India. Cost-utility analysis constituted the predominant methodological approach, complemented by one cost-effectiveness analysis and one budget impact analysis. All studies adopted Markov modeling frameworks, with time horizons ranging from one year to a lifetime.

Economic assessments were conducted from various perspectives, including payer, healthcare system, and societal viewpoints. Most state-transition probabilities were derived from phase III clinical trial data, ensuring consistency in clinical inputs across models. The evaluations encompassed a broad spectrum of tumor types, including breast cancer, prostate cancer, multiple myeloma, and other solid tumors. For analytical comparability, all monetary values were standardized to U.S. dollars.

Given the inherent context-dependence of cost-effectiveness analyses, the results were synthesized by geographic region to account for differences in willingness-to-pay (WTP) thresholds, healthcare financing structures, and pricing policies.

In the United States, Canada, and several European countries (Austria, Sweden, Switzerland, and the Czech Republic), denosumab consistently emerged as a cost-effective option relative to zoledronic acid. Across these analyses, drug acquisition cost was the dominant cost driver; however, the higher upfront expense of denosumab was partially offset by substantial reductions in SRE-related resource use, including radiation therapy, surgery, and hospitalization. Reported ICERs typically ranged from USD 15, 000 to 109, 666 per QALY gained, values that generally fall within or below commonly applied regional WTP thresholds (e.g., USD 50, 000–100, 000 per QALY). These findings collectively indicate that, in resource-rich settings with higher WTP capacities, denosumab is economically attractive.

Economic conclusions varied more widely across Asian health systems due to divergent drug pricing mechanisms and differences in healthcare affordability.

In China, denosumab demonstrated favorable cost-effectiveness in scenarios influenced by negotiated pricing or patient assistance programs. For example, Li et al. (38) identified denosumab as a dominant strategy, offering both lower costs and higher effectiveness, while Chen et al. (37) reported ICER values well below the WHO-recommended threshold of 1–3 times GDP per capita.

In contrast, India presented a markedly different economic profile. Wadhwa et al. (34) estimated an ICER of USD 485, 558 per QALY, substantially exceeding local WTP thresholds and reflecting the wide cost differential between originator denosumab and low-cost generic zoledronic acid. These findings highlight the challenge of adopting high-priced biologics in resource-limited environments.

Although denosumab consistently exhibits superior clinical performance across tumor types, its economic value remains strongly contingent upon local pricing negotiations, healthcare system affordability, and regional WTP benchmarks. These contextual factors largely explain the geographic variability observed across the included pharmacoeconomic evaluations and underscore the importance of tailoring reimbursement decisions to country-specific economic constraints and policy priorities.

## 4 Discussion

This study systematically evaluated the clinical efficacy, safety, and economic evidence of Dmab and ZA in patients with bone metastases from solid tumors and multiple myeloma. Synthesizing the available data, moderate-certainty evidence suggests that Dmab likely provides superior efficacy in delaying both the first SRE and subsequent SREs. Regarding survival outcomes, the analysis found no statistically significant differences, indicating with moderate certainty that the two agents confer comparable effects on OS and PFS. Additionally, low-certainty evidence suggests that Dmab may have a more favorable safety profile, with a lower incidence of adverse events, although this advantage must be balanced against its higher acquisition cost. Consequently, economic evaluations indicate that the cost-effectiveness of Dmab is highly context-dependent and varies substantially across healthcare systems.

Regarding clinical efficacy, current evidence indicates that Dmab confers superior performance compared to ZA in delaying both first and subsequent SREs, a finding that aligns with outcomes from prior large-scale phase III clinical trials and meta-analyses (8, 14, 19).

The distinct efficacy profiles of the two agents are rooted in their divergent mechanisms of action. Zoledronic acid, a bisphosphonate, binds avidly to the bone mineral matrix and inhibits osteoclast farnesyl pyrophosphate synthase upon internalization, thereby inducing apoptosis (39). However, its efficacy relies on uptake during active bone resorption. In contrast, denosumab is a monoclonal antibody that targets RANKL, a key mediator of osteoclast formation, function, and survival. By sequestering RANKL, denosumab mimics the physiological action of osteoprotegerin (OPG), effectively arresting the ‘vicious cycle’ of bone destruction driven by tumor-derived cytokines (e.g., PTHrP) (40, 41). This mechanism is particularly critical in osteolytic conditions (e.g., multiple myeloma, breast cancer) where osteoclast overactivation is the primary driver. However, it is also relevant in osteoblastic or mixed lesions (e.g., prostate cancer), as increased bone resorption is often a prerequisite for the subsequent unregulated bone formation, making RANKL a universal therapeutic target across metastatic phenotypes (42).

Although this meta-analysis did not demonstrate a statistically significant improvement in OS for either agent, the clinical value of delaying SREs extends beyond conventional survival metrics. SREs, including pathological fractures and spinal cord compression, act as sentinel events that trigger functional decline, immobility, and nutritional compromise (7). By preventing such events, bone-modifying agents help preserve patient independence and physiological reserve. While not directly antineoplastic, effective bone protection can stabilize the overall clinical course, potentially enabling patients to maintain the performance status necessary to tolerate subsequent systemic therapies. Consequently, preservation of quality of life and functional mobility constitutes a critical therapeutic objective, independent of OS outcomes.

The safety analysis suggested that Dmab may offer a more favorable tolerability profile; however, the interpretation of these findings requires careful consideration of potential detection and reporting biases. Although low-certainty evidence indicated that Dmab was associated with a reduced risk of any adverse event (OR = 0.70, P = 0.04), a difference that may contribute to better treatment persistence, the observed toxicity patterns largely reflect the distinct pharmacokinetic and pharmacodynamic characteristics of the two agents.

The significantly lower risk of nephrotoxicity with Dmab (OR = 0.65, P = 0.02) is biologically consistent with their divergent elimination pathways. ZA undergoes renal clearance and can accumulate within renal tubular epithelial cells, leading to direct cytotoxicity (43). In contrast, Dmab is metabolized via the reticuloendothelial system and does not rely on renal excretion, thereby imposing minimal renal burden. Dmab also demonstrated clear advantages in acute-phase reactions, showing significantly lower risks of fever (OR = 0.70) and acute-phase responses (OR = 0.35) compared with ZA. These events are attributable to bisphosphonate-induced γδ T-cell activation and subsequent cytokine release (e.g., IL-6, TNF-α), a mechanism not triggered by RANKL inhibition.

Despite the apparent superiority of Dmab, some safety differences may be partially driven by systematic detection biases. Standard ZA administration protocols mandate serum creatinine assessment prior to each infusion, increasing the probability of detecting transient renal abnormalities. In contrast, Dmab is typically monitored less intensively, which may result in under-ascertainment of mild renal signals. Similarly, the lack of a statistically significant difference in ONJ risk (OR = 0.99) should be interpreted cautiously, as diagnostic criteria varied across studies, ranging from clinical examination alone to mandatory radiographic confirmation.

Additionally, treatment adherence patterns differ substantially between the two drugs. The convenience of subcutaneous Dmab generally promotes higher persistence, whereas the infusion burden associated with intravenous ZA contributes to earlier discontinuation. This differential exposure creates an imbalance in the time at risk for cumulative toxicities, potentially influencing long-term safety profiles.

Collectively, while the current evidence suggests that Dmab may confer certain safety advantages, these findings are tempered by variability in monitoring intensity, diagnostic practices, and treatment persistence. Future trials employing harmonized safety assessment protocols are needed to clarify the true comparative toxicity profiles of Dmab and ZA.

Economic evaluations suggest that, although Dmab confers clear clinical benefits in delaying SREs, its cost-effectiveness is highly context-dependent and demonstrates considerable heterogeneity across healthcare systems. This variability is primarily driven by geographic region, differences in model assumptions, and the choice of willingness-to-pay thresholds. Detailed cost-structure analyses indicate that the relatively high unit price of Dmab constitutes the main determinant of its higher total treatment cost relative to ZA. While clinical data indicate that Dmab likely reduces both first and subsequent SREs and may offer a more favorable safety profile, the resultant savings in healthcare utilization only partially offset the higher acquisition costs. Furthermore, cost-effectiveness outcomes differ markedly across countries: model-based analyses in the United States, Canada, and several European nations report incremental costs per QALY gained ranging from approximately $15, 000 to $70, 000, supporting the classification of Dmab as cost-effective in these regions. By contrast, in resource-limited settings or countries with divergent drug pricing structures, ZA may remain the more economically viable option. These findings underscore the necessity of considering local pricing, healthcare utilization patterns, and payer perspectives when interpreting the economic value of Dmab.

A major limitation of the included economic evaluations concerns the restricted transferability of their model structures and input parameters. Most analyses employed Markov models that relied heavily on transition probabilities derived from phase III RCTs. Although RCTs generate internally robust efficacy estimates, their participant populations typically diverge from real-world patients in comorbidity profiles, prior treatment exposure, and adherence behaviors. As a result, the progression rates embedded within these models may not accurately replicate routine clinical practice.

Furthermore, generalizability is constrained by pronounced cross-country differences in healthcare cost structures. Findings such as negative ICERs or dominance should therefore be interpreted strictly within the context of the local pricing and reimbursement environment.

Across the included studies, sensitivity analyses consistently identified drug acquisition cost as the predominant driver of economic uncertainty. Inpatient costs associated with managing SREs, including radiation therapy and orthopedic surgery, also exerted a substantial influence on ICER variability. This pattern indicates that in healthcare systems with high hospitalization costs, the economic value of denosumab is magnified through its superior capacity to prevent SREs. Conversely, in settings characterized by relatively low inpatient costs but substantial disparities in drug pricing, the economic advantage of denosumab diminishes.

This study systematically evaluated the efficacy, safety, and pharmacoeconomic profile of Dmab versus ZA through a comprehensive systematic review and meta-analysis; however, several limitations should be acknowledged. First, despite the application of rigorous quality assessment, residual selection bias and publication bias may persist among the included studies. Second, substantial heterogeneity exists across economic evaluations in terms of model structures, parameter selection, and cost assumptions, which limits the direct comparability of their results. Moreover, most included studies focused primarily on clinical endpoints such as SRE incidence, with relatively limited integration of patient-reported outcomes (PROs) and health-related quality of life measures.

Additionally, the interpretation of our findings must consider the rapidly evolving therapeutic landscape, particularly in multiple myeloma. Several included studies (14, 19, 22) were conducted before the widespread adoption of contemporary systemic therapies such as next-generation proteasome inhibitors, monoclonal antibodies (e.g., daratumumab), bispecific antibodies, and CAR-T cell therapies (44, 45). These advancements have improved disease control and likely reduced baseline SRE rates relative to historical cohorts. Therefore, the absolute benefits of bone-modifying agents observed in earlier studies may not fully generalize to patients receiving modern induction regimens or novel immunotherapies. Future research should assess the comparative effectiveness of Dmab versus ZA within contemporary treatment paradigms to determine whether the relative benefits observed in this meta-analysis persist among patients achieving deeper responses.

Finally, given the evolving landscape, the choice of bone-modifying agent may have implications beyond structural bone protection, particularly with respect to drug resistance and the bone marrow microenvironment. In conditions such as monoclonal gammopathy of undetermined significance (MGUS) and multiple myeloma, drug resistance is increasingly recognized as a dynamic process shaped by the marrow niche. Stromal cells, osteoclasts, and immune regulatory cells provide survival signals that protect malignant plasma cells from cytotoxic therapies, a phenomenon known as cell-adhesion-mediated drug resistance (CAM-DR) (45). The RANKL pathway, central to osteoclast activation, is a key contributor to this permissive microenvironment (46). Consequently, inhibition of this pathway with denosumab or suppression of osteoclast function via zoledronic acid may modulate marrow niches, cytokine gradients, and the osteoclast–osteoblast balance, thereby reducing microenvironmental protection. Future studies should investigate whether microenvironment-directed interventions can synergize with anti-myeloma therapies to delay progression in early disease or overcome resistance in relapsed/refractory settings.

Beyond biological mechanisms, future clinical research should also more fully incorporate PROs, pain control, and functional status as key endpoints, while integrating real-world evidence to enhance the external validity of study findings. Moreover, considering the economic heterogeneity identified in this study, future evaluations should explicitly assess the market entry of denosumab biosimilars following patent expirations. The availability of lower-cost biosimilars has the potential to fundamentally alter the cost-effectiveness landscape, potentially making RANKL inhibition a viable standard of care even in resource-constrained healthcare settings.

## 5 Conclusion

This systematic review and meta-analysis indicates that, among patients with bone metastases from solid tumors or multiple myeloma, denosumab probably provides superior efficacy over zoledronic acid in delaying the onset of both first and subsequent SREs, with the advantage being most pronounced in solid tumors. In contrast, the current evidence suggests no meaningful efficacy difference between the two agents in multiple myeloma, underscoring the influence of underlying tumor biology on treatment performance. With respect to safety, denosumab appears to offer better renal tolerability, consistent with its distinct pharmacologic mechanism. No statistically significant differences were detected for overall survival or progression-free survival.

Pharmacoeconomic evidence further indicates that the cost-effectiveness of denosumab is highly context-specific, shaped primarily by regional willingness-to-pay thresholds, healthcare financing structures, and drug pricing policies. Consequently, while denosumab remains an important therapeutic option with demonstrated clinical advantages, its optimal use should be tailored according to tumor type, patient safety profiles, and the economic feasibility within a given healthcare system.

## Data Availability

The original contributions presented in the study are included in the article/**Supplementary Material**. Further inquiries can be directed to the corresponding authors.
