# Supplementary material for: Efficacy, safety and economy of denosumab and zoledronic acid in the treatment of bone metastases of solid tumors and multiple myeloma: a systematic review and meta-analysis
Source: Front Oncol. 2026 Jan 6;15:1747354. doi: 10.3389/fonc.2025.1747354 (PMC12815878; doi:10.3389/fonc.2025.1747354)
Supplement: Supplementary file 1 [file Table1.docx]

**Supplementary** **Material**

[Table S1 The search strategy of database search 2](#_Toc216257581)

[Table S2. Quality Assessment of Cohort Studies 6](#_Toc216257582)

[Table S3.Summary of missing outcome data and handling strategies for the included clinical studies 7](#_Toc216257583)

[Table S4. Results of the Meta-analysis comparison of the incidence of specific adverse events between denosumab and zoledronic acid 8](#_Toc216257584)

[Table S5. Leave-One-Out Sensitivity Analysis for Time to first SRE 9](#_Toc216257585)

[Table S6.Leave-One-Out Sensitivity Analysis for Time to first and subsequent SREs 9](#_Toc216257586)

[Table S7.Leave-One-Out Sensitivity Analysis for Overall survival 9](#_Toc216257587)

[Table S8.Leave-One-Out Sensitivity Analysis for Progression-free survival 9](#_Toc216257588)

[Table S9.Leave-One-Out Sensitivity Analysis for Incidence of any adverse events 10](#_Toc216257589)

[Table S10.Leave-One-Out Sensitivity Analysis for Incidence of serious adverse events 10](#_Toc216257590)

[Table S11. GRADE Evidence Quality Assessment Summary 11](#_Toc216257591)

[PRISMA checklist 12](#_Toc216257592)

# Table S1 The search strategy of database search

| **PubMed** | | |
| --- | --- | --- |
| ID | Search Terms | Results |
| #1 | "Denosumab"[Mesh] | 2733 |
| #2 | (((Denosumab[Title/Abstract]) OR (Prolia[Title/Abstract])) OR (Xgeva[Title/Abstract])) OR (AMG 162[Title/Abstract]) | 4975 |
| #3 | ("Denosumab"[Mesh]) OR ((((Denosumab[Title/Abstract]) OR (Prolia[Title/Abstract])) OR (Xgeva[Title/Abstract])) OR (AMG 162[Title/Abstract])) | 5224 |
| #4 | "Zoledronic Acid"[Mesh] | 4329 |
| #5 | ((((((Zoledronic Acid[Title/Abstract]) OR (Zoledronic Acid[Title/Abstract])) OR (CGP 42446[Title/Abstract])) OR (CGP 42446[Title/Abstract])) OR (CGP) | 4739 |
| #6 | ("Zoledronic Acid"[Mesh]) OR (((((((Zoledronic Acid[Title/Abstract]) OR (Zoledronic Acid[Title/Abstract])) OR (CGP 42446[Title/Abstract])) OR (CGP | 6234 |
| #7 | "Breast Neoplasms"[Mesh] | 366105 |
| #8 | "Bone Neoplasms"[Mesh] | 140954 |
| #9 | ("Breast Neoplasms"[Mesh]) AND ("Bone Neoplasms"[Mesh]) | 7672 |
| #10 | "Prostatic Neoplasms"[Mesh] | 161,920 |
| #11 | "Prostatic Neoplasms"[Mesh] AND ("Bone Neoplasms"[Mesh]) | 6744 |
| #12 | "Multiple myeloma"[Mesh] | 51676 |
| #13 | "Multiple myeloma"[Mesh]AND ("Bone Neoplasms"[Mesh]) | 2211 |
| #14 | ((("Denosumab"[MeSH Terms] OR ("Denosumab"[Title/Abstract] OR "Prolia"[Title/Abstract] OR "Xgeva"[Title/Abstract] OR "amg 162"[Title/Abstract])) AND ("Zoledronic Acid"[MeSH Terms] OR ("Zoledronic Acid"[Title/Abstract] OR "Zoledronic Acid"[Title/Abstract] OR "cgp 42446"[Title/Abstract] OR "cgp 42446"[Title/Abstract] OR "cgp 42446a"[Title/Abstract] OR "zoledronic acid anhydrous"[Title/Abstract] OR (("2"[All Fields] AND "Imidazol-1-yl"[All Fields]) AND ("1 hydroxyethylidene 1"[All Fields] AND "1 bisphosphonic acid"[Title/Abstract])))) AND ("Breast Neoplasms"[MeSH Terms] AND "Bone Neoplasms"[MeSH Terms])) OR ("Prostatic Neoplasms"[Mesh]AND ("Bone Neoplasms"[Mesh]))) AND ("Multiple myeloma"[Mesh]AND ("Bone Neoplasms"[Mesh])) | 96 |
| **Web of Science** | | |
| ID | Search Terms | Results |
| #1 | Denosumab (Topic) OR Prolia (Topic) OR Xgeva (Topic) OR AMG 162 (Topic) and Preprint Citation Index (Exclude – Database) | 9191 |
| #2 | Zoledronic Acid (Topic) OR Zometa (Topic) OR CGP 42446 (Topic) OR CGP 42446A (Topic) OR Zoledronic Acid Anhydrous (Topic) OR 2-(Imidazol-1-yl)-1-hydroxyethylidene-1,1-bisphosphonic acid (Topic) and Preprint Citation Index (Exclude – Database) | 15344 |
| #3 | Prostate cancer (Topic) and Bone metastasis (Topic) | 8822 |
| #4 | Breast cancer (Topic) and Bone metastasis (Topic) | 10377 |
| #5 | Multiple myeloma (Topic) and Bone metastasis (Topic) | 1358 |
| #6 | ((((TS=(Denosumab (Topic) OR Prolia (Topic) OR Xgeva (Topic) OR AMG 162 (Topic) and Preprint Citation Index (Exclude – Database) )) AND TS=(Zoledronic Acid (Topic) OR Zometa (Topic) OR CGP 42446 (Topic) OR CGP 42446A (Topic) OR Zoledronic Acid Anhydrous (Topic) OR 2-(Imidazol-1-yl)-1-hydroxyethylidene-1,1-bisphosphonic acid (Topic) and Preprint Citation Index (Exclude – Database) )) AND #3) AND#4) OR #5 and Open Access | 557 |
| **Embase** | | |
| ID | Search Terms | Results |
| #1 | 'denosumab'/exp | 16511 |
| #2 | denosumab:ab,ti OR zadenvi:ab,ti OR yaxwer:ab,ti OR xbryk:ab,ti OR hs20090:ab,ti OR fks518:ab,ti OR 'denosumab bbdz':ab,ti OR amgiva:ab,ti OR 'amg 162':ab,ti | 9959 |
| #3 | #1 OR #2 | 17060 |
| #4 | 'zoledronic acid'/exp | 23417 |
| #5 | 'zoledronic acid':ab,ti OR zomera:ab,ti OR zoledon:ab,ti OR zol446:ab,ti OR nordeloz:ab,ti OR cgp42446a:ab,ti OR axizoledron:ab,ti OR ('[1 hydroxy 2':ab,ti AND '1h imidazol 1 yl':ab,ti AND '1 phosphonoethyl] phosphonic acid':ab,ti) OR aclasta:ab,ti | 9188 |
| #6 | #4 OR #5 | 23818 |
| #7 | 'bone metastasis'/exp | 71180 |
| #8 | 'bone metastasis':ab,ti OR 'skeleton metastasis':ab,ti OR 'osseous metastasis':ab,ti OR 'bone metastatic tumor':ab,ti | 17337 |
| #9 | #7 OR #8 | 74410 |
| #10 | 'prostate cancer'/exp | 312816 |
| #11 | 'prostate cancer':ab,ti OR 'prostate malignant tumour':ab,ti OR 'prostate malignancy':ab,ti OR 'prostate carcinogenesis':ab,ti OR 'malignant prostate tumor':ab,ti OR 'cancer, prostate':ab,ti OR 'carcinogenesis of the prostate':ab,ti OR 'malignancies of the prostate':ab,ti | 254474 |
| #12 | #10 OR #11 | 350033 |
| #13 | 'breast cancer'/exp | 685029 |
| #14 | 'breast cancer':ab,ti OR 'mammary malignancies':ab,ti OR 'malignant tumor of the breast':ab,ti OR 'cancer of the mammary gland':ab,ti OR 'ca breast':ab,ti OR 'breast malignancy':ab,ti OR 'breast gland cancer':ab,ti | 578720 |
| #15 | #13 OR #14 | 792011 |
| #16 | 'multiple myeloma'/exp | 125019 |
| #17 | 'multiple myeloma':ab,ti OR myelomatosis:ab,ti OR 'plasma cell myeloma':ab,ti OR 'kahler disease':ab,ti OR 'morbus kahler':ab,ti | 101933 |
| #18 | #16 OR #17 | 134449 |
| #19 | #9 AND #12 | 17876 |
| #20 | #9 AND #15 | 17062 |
| #21 | #9 AND #18 | 2507 |
| #22 | #3 AND #6 | 5574 |
| #23 | #22 AND #20 OR #21 | 803 |
| **CBM** | | |
| ID | Search Terms | Results |
| #1 | ("Bone Neoplasms"[Unweighted: Explode] OR "Bone Metastasis"[All Fields] OR "Multiple Myeloma"[Unweighted: Explode]) | 31329 |
| #2 | ("Denosumab"[All Fields] OR "Xgeva"[All Fields] OR "Prolia"[All Fields]) | 286 |
| #3 | ("Zoledronic Acid"[All Fields] OR "Zometa"[All Fields] | 2905 |
| #4 | #1 AND #2 AND #3 | 2 |
| **CNKI** | | |
| ID | Search Terms | Results |
| #1 | (Subject: "Bone Metastasis" OR "Breast Cancer" OR "Prostate Cancer" OR "Multiple Myeloma") | 7724 |
| #2 | (Subject: "Zoledronic Acid" OR "Zoledronate") | 4132 |
| #3 | Subject: "Denosumab" OR "Xgeva" OR "Prolia" OR "Bolujia") | 485 |
| #4 | #1 AND #2 AND #3 | 5 |

#

# Table S2. **Quality Assessment of Cohort Studies**

| Study | ① | ② | ③ | ④ | ⑤ | ⑥ | ⑦ | ⑧ | NOS |
| --- | --- | --- | --- | --- | --- | --- | --- | --- | --- |
| He2022 | 1 | 1 | 1 | 1 | 1 | 1 | 1 | 1 | 8 |
| Zhang 2023 | 1 | 1 | 1 | 1 | 1 | 1 | 1 | 1 | 8 |
| He 2024 | 1 | 1 | 1 | 0 | 1 | 1 | 1 | 1 | 7 |
| Huang 2024 | 1 | 1 | 1 | 0 | 2 | 1 | 1 | 1 | 8 |
| Scafetta 2025 | 1 | 1 | 1 | 0 | 2 | 1 | 1 | 0 | 7 |
| Note:① Representativeness of the Exposed Cohort (1 point);② Selection of the Non-Exposed Cohort (1 point);  ③ Ascertainment of Exposure (1 point);④ Demonstration That Outcome of Interest Was Not Present at Start of Study (1 point);⑤ Comparability of Cohorts on the Basis of the Design or Analysis (2 points);⑥ Assessment of Outcome (1 point);⑦ Was Follow-Up Long Enough for Outcomes to Occur (1 point);⑧ Adequacy of Follow-Up of Cohorts (1 point). | | | | | | | | | |

# Table S3.Summary of missing outcome data and handling strategies for the included clinical studies

| Study | Research type | Outcome indicators | Missing Outcomes | Reason for Missingness & Handling Strategy |
| --- | --- | --- | --- | --- |
| Stopeck, A.2010 | RCT | ①②③④⑤⑥ | None | Complete data were available for all pre-specified outcomes. |
| Fizazi.2011 | RCT | ①②③④⑤⑥ | None | Complete data were available for all pre-specified outcomes. |
| Henry, D.H.2011 | RCT | ①②③④⑤⑥ | None | Complete data were available for all pre-specified outcomes. |
| Vadhan-Raj, S.2012 | RCT | ①② | ③④⑤⑥ | Not reported in the primary publication. The study focused on SRE endpoints. |
| Rajie.2018 | RCT | ①②③④⑤⑥ | None | Complete data were available for all pre-specified outcomes. |
| He. 2022 | Retrospective cohort study | ⑤ | ①②③④⑥ | Not Reported. The study focused primarily on overall safety. Data were not available for efficacy endpoints or SAEs. Excluded from respective analyses. |
| Zhang. 2023 | Retrospective cohort study | ⑥ | ①②③④⑤ | Not Reported. Outcomes were limited to adverse events. Excluded from efficacy meta-analyses. |
| He. 2024 | Retrospective cohort study | ⑤ | ①②③④⑥ | Not Reported. The study reported overall adverse events but lacked data on time-to-event efficacy outcomes and serious adverse events. |
| Huang. 2024 | Retrospective cohort study | ⑤⑥ | ①②③④ | Not reported. Data format unavailable. Efficacy was reported as incidence rates (OR) rather than time-to-event data (HR). Detailed survival data were not provided. |
| Scafetta.2025 | Retrospective cohort study | ① | ②③④⑤⑥ | Not Reported. The study focused specifically on Time to first SRE. Safety data were not stratified by treatment group or were not reported in sufficient detail for extraction. |
| Note: ①Time to first SRE②Time to first and subsequent SREs ③Overall Survival (OS) ④Progression-Free Survival(PFS) ⑤Overall incidence of Adverse Events ⑥Incidence of Serious Adverse Events (SAEs)  Handling Strategy: For all missing data listed above, corresponding authors were contacted via email. As no additional unpublished data were obtained within the revision timeframe, analyses were restricted to the available data reported in the original articles. | | | | |

# Table S4. Results of the Meta-analysis comparison of the incidence of specific adverse events between denosumab and zoledronic acid

| Adverse event | Included literature | Events/Total | | Results of heterogeneity test | | Effect model | Results of Meta-analysis | |
| --- | --- | --- | --- | --- | --- | --- | --- | --- |
|  |  | Dmab | ZA | I^2^（%） | P |  | OR（95%Cl） | P |
| Nephrotoxicity | [14,19,20,25-27] | 486/3814 | 638/3819 | 77 | 0.002 | Random effects | 0.65[0.45-0.94] | 0.02 |
| nauseous | [14,19,20,22,24] | 1152/3721 | 1171/3718 | 40 | 0.15 | Random effects | 0.98[0.85-1.12] | 0.73 |
| Anemia | [14,19,20,22] | 1082/3691 | 1152/3688 | 58 | 0.07 | Random effects | 0.91[0.78-1.07] | 0.25 |
| Dyspnea | [19,22] | 451/1898 | 524/1891 | 0 | 0.33 | Random effects | 0.81[0.70-0.94] | 0.005 |
| Fatigue | [14,19] | 478/1821 | 442/1823 | 37 | 0.21 | Random effects | 1.11[0.92-1.34] | 0.28 |
| Constipation | [14,19,20,22] | 808/3691 | 875/3688 | 0 | 0.63 | Random effects | 0.90[0.81-1.01] | 0.06 |
| vomit | [19,22] | 398/1898 | 421/1891 | 21 | 0.26 | Random effects | 0.93[0.78-1.10] | 0.40 |
| Back and waist pain | [14,19,20,22] | 913/3691 | 925/3688 | 41 | 0.16 | Random effects | 0.98[0.85-1.13] | 0.78 |
| Weakness | [14,19,20,22] | 923/3691 | 944/3688 | 56 | 0.08 | Random effects | 0.97[0.83-1.14] | 0.69 |
| Anorexia | [14,19,20] | 593/2673 | 606/2673 | 63 | 0.07 | Random effects | 0.98[0.79-1.21] | 0.84 |
| Fever | [19,20,22,26] | 489/2814 | 646/2809 | 37 | 0.19 | Random effects | 0.70[0.59-0.84] | 0.0001 |
| Acute phase response | [19,22] | 167/1898 | 404/1891 | 67 | 0.08 | Random effects | 0.35[0.29-0.43] | ＜0.00001 |
| Osteonecrosis of the jaw | [14,19,20,24,26] | 48/2767 | 48/2769 | 28 | 0.24 | Random effects | 0.99[0.58-1.68] | 0.96 |
| Newly diagnosed malignant tumor | [14,19,22] | 28/2841 | 18/2836 | 0 | 0.72 | Random effects | 1.56[0.86-2.84] | 0.15 |
| Hypocalcemia | [14,20,26] | 248/1861 | 224/1861 | 94 | 0.00001 | Random effects | 1.35[0.50-3.62] | 0.55 |
| Bone pain | [14,22] | 421/1963 | 483/1958 | 67 | 0.08 | Random effects | 0.83[0.64-1.08] | 0.17 |

#

# Table S5. Leave-One-Out Sensitivity Analysis for Time to first SRE

| **Study Excluded** | **Pooled HR (95% CI)** | P-value | **I²** |
| --- | --- | --- | --- |
| None(Original Result) | 0.85[ 0.79-0.93] | 0.0001 | 20% |
| Stopeck, A.2010 | 0.86[ 0.78-0.95] | 0.004 | 34% |
| Scafetta.2025 | 0.86[0.79-0.94] | 0.001 | 26% |
| Fiazi , K.2011 | 0.86 [ 0.78-0.95] | 0.004 | 34% |
| Rajie.2018 | 0.82 [ 0.75-0.89] | <0.00001 | 0% |
| Henry, D.H.2011 | 0.86 [ 0.77-0.95] | 0.003 | 39% |

# Table S6.Leave-One-Out Sensitivity Analysis for Time to first and subsequent SREs

| **Study Excluded** | **Pooled HR (95% CI)** | P-value | **I²** |
| --- | --- | --- | --- |
| None(Original Result) | 0.86[ 0.76-0.97] | 0.02 | 65% |
| Stopeck, A.2010 | 0.89 [ 0.78-1.02] | 0.11 | 63% |
| Fiazi,K.2011 | 0.87[0.74-1.03] | 0.11 | 74% |
| Rajie.2018 | 0.81 [ 0.74-0.88] | <0.00001 | 0% |
| Henry, D.H.2011 | 0.87 [ 0.73-1.02] | 0.09 | 76% |

# Table S7.Leave-One-Out Sensitivity Analysis for Overall survival

| **Study Excluded** | **Pooled HR (95% CI)** | P-value | **I²** |
| --- | --- | --- | --- |
| None(Original Result) | 0.97[ 0.91-1.05] | 0.49 | 0% |
| Stopeck, A.2010 | 0.98 [ 0.90-1.06] | 0.65 | 0% |
| \| Scafetta.2025 \| \| --- \| | 0.97[0.90-1.05] | 0.51 | 0% |
| Fiazi , K.2011 | 0.95[0.87-1.04] | 0.23 | 0% |
| Rajie.2018 | 0.98 [ 0.91-1.06] | 0.63 | 0% |
| Henry, D.H.2011 | 0.98 [ 0.90-1.07] | 0.71 | 0% |

# Table S8.Leave-One-Out Sensitivity Analysis for Progression-free survival

| **Study Excluded** | **Pooled HR (95% CI)** | P-value | **I²** |
| --- | --- | --- | --- |
| None(Original Result) | 0.99[ 0.93-1.06] | 0.86 | 24% |
| Stopeck, A.2010 | 0.99 [ 0.90-1.09] | 0.84 | 46% |
| \| Scafetta.2025 \| \| --- \| | 0.98 [ 0.91-1.07] | 0.71 | 44% |
| Fiazi , K.2011 | 0.97[0.90-1.05] | 0.49 | 25% |
| Rajie.2018 | 1.02 [ 0.96-1.08] | 0.56 | 0% |
| Henry, D.H.2011 | 0.99 [ 0.90-1.09] | 0.80 | 46% |

# Table S9.Leave-One-Out Sensitivity Analysis for Incidence of any adverse events

| **Study Excluded** | **Pooled OR (95% CI)** | P-value | **I²** |
| --- | --- | --- | --- |
| None(Original Result) | 0.70[ 0.50-0.98] | 0.04 | 40% |
| Stopeck, A.2010 | 0.69 [ 0.45-1.06] | 0.09 | 48% |
| Huang Xiang.2024 | 0.80 [ 0.63-1.03] | 0.08 | 0% |
| He Qingan.2024 | 0.74 [ 0.55-1.01] | 0.06 | 33% |
| Fiazi,K.2011 | 0.64[0.43-0.94] | 0.02 | 42% |
| He Qingliu.2022 | 0.70[0.49-1.00] | 0.05 | 50% |
| Rajie.2018 | 0.65 [ 0.41-1.04] | 0.07 | 60% |
| Henry, D.H.2011 | 0.62 [ 0.40-0.92] | 0.03 | 52% |

# Table S10.Leave-One-Out Sensitivity Analysis for Incidence of serious adverse events

| **Study Excluded** | **Pooled OR (95% CI)** | P-value | **I²** |
| --- | --- | --- | --- |
| None(Original Result) | 0.96[ 0.87-1.07] | 0.45 | 14% |
| Stopeck, A.2010 | 0.98[ 0.85-1.12] | 0.73 | 27% |
| Huang Xiang.2024 | 0.96[ 0.87-1.06] | 0.42 | 14% |
| Fiazi,K.2011 | 0.91[0.82-1.01] | 0.09 | 0% |
| Zhang Tongsong .2023 | 0.96[0.86-1.08] | 0.53 | 26% |
| Rajie.2018 | 0.96 [ 0.83-1.11] | 0.62 | 31% |
| Henry, D.H.2011 | 0.99 [ 0.88-1.11] | 0.88 | 9% |

# Table S11. GRADE Evidence Quality Assessment Summary

| **Outcome** | **No. of studies** | **Quality Assessment (Downgrade Factors)** | | | | | **I²** | **Effect (95% CI)** | **Quality** |
| --- | --- | --- | --- | --- | --- | --- | --- | --- | --- |
|  |  | **Risk of bias** | **Inconsistency** | **Indirectness** | **Imprecision** | **Publication bias** |  |  |  |
| Time to first SRE | 5 | -1¹ | 0 | 0 | 0 | 0 | 20% | HR 0.85 [0.79-0.93] | ⊕⊕⊕○ Moderate |
| Time to first and subsequent SREs | 4 | 0 | -1² | 0 | 0 | 0 | 65% | HR 0.86 [0.76-0.97] | ⊕⊕⊕○ Moderate |
| Overall survival | 5 | -1¹ | 0 | 0 | 0 | 0 | 0% | HR 0.97 [0.91-1.05] | ⊕⊕⊕○ Moderate |
| Progression-free survival | 5 | -1¹ | 0 | 0 | 0 | 0 | 28% | HR 0.99 [0.93-1.06] | ⊕⊕⊕○ Moderate |
| Any adverse events | 7 | -2³ | 0 | 0 | 0 | 0 | 40% | OR 0.70 [0.50-0.98] | ⊕⊕○○ Low |
| Serious adverse events | 6 | -2³ | 0 | 0 | 0 | 0 | 14% | OR 0.96 [0.87-1.07] | ⊕⊕○○ Low |

Note:

¹ Downgraded one level (-1): Inclusion of one observational study with potential confounding factors.

² Downgraded one level (-1): Substantial heterogeneity (I² = 65%).

³ Downgraded two levels (-2): Inclusion of multiple observational studies (≥2) with potential confounding factors.

*Abbreviations: HR, hazard ratio; OR, odds ratio; CI, confidence interval; RCT, randomized controlled trial; Obs, observational study; SRE, skeletal-related event.*

*GRADE Working Group grades of evidence: High (⊕⊕⊕⊕) = Further research is very unlikely to change our confidence in the estimate of effect; Moderate (⊕⊕⊕○) = Further research is likely to have an important impact on our confidence in the estimate of effect and may change the estimate; Low (⊕⊕○○) = Further research is very likely to have an important impact on our confidence in the estimate of effect and is likely to change the estimate; Very low (⊕○○○) = We are very uncertain about the es*

# PRISMA checklist

| **Section and Topic** | **Item #** | **Checklist item** | **Location where item is reported** |
| --- | --- | --- | --- |
| **TITLE** | | |  |
| Title | 1 | Identify the report as a systematic review. | Title |
| **ABSTRACT** | | |  |
| Abstract | 2 | See the PRISMA 2020 for Abstracts checklist. | Abstract |
| **INTRODUCTION** | | |  |
| Rationale | 3 | Describe the rationale for the review in the context of existing knowledge. | Introduction |
| Objectives | 4 | Provide an explicit statement of the objective(s) or question(s) the review addresses. | Introduction |
| **METHODS** | | |  |
| Eligibility criteria | 5 | Specify the inclusion and exclusion criteria for the review and how studies were grouped for the syntheses. | Methods  (Section 2.2) |
| Information sources | 6 | Specify all databases, registers, websites, organisations, reference lists and other sources searched or consulted to identify studies. Specify the date when each source was last searched or consulted. | Methods  (Section 2.1) |
| Search strategy | 7 | Present the full search strategies for all databases, registers and websites, including any filters and limits used. | Methods  (Section 2.1) |
| Selection process | 8 | Specify the methods used to decide whether a study met the inclusion criteria of the review, including how many reviewers screened each record and each report retrieved, whether they worked independently, and if applicable, details of automation tools used in the process. | Methods  (Section 2.3) |
| Data collection process | 9 | Specify the methods used to collect data from reports, including how many reviewers collected data from each report, whether they worked independently, any processes for obtaining or confirming data from study investigators, and if applicable, details of automation tools used in the process. | Methods  (Section 2.3) |
| Data items | 10a | List and define all outcomes for which data were sought. Specify whether all results that were compatible with each outcome domain in each study were sought (e.g. for all measures, time points, analyses), and if not, the methods used to decide which results to collect. | Methods  (Section 2.3) |
|  | 10b | List and define all other variables for which data were sought (e.g. participant and intervention characteristics, funding sources). Describe any assumptions made about any missing or unclear information. | Table1 Table2 |
| Study risk of bias assessment | 11 | Specify the methods used to assess risk of bias in the included studies, including details of the tool(s) used, how many reviewers assessed each study and whether they worked independently, and if applicable, details of automation tools used in the process. | Methods  (Section 2.4) |
| Effect measures | 12 | Specify for each outcome the effect measure(s) (e.g. risk ratio, mean difference) used in the synthesis or presentation of results. | Methods  (Section 2.5) |
| Synthesis methods | 13a | Describe the processes used to decide which studies were eligible for each synthesis (e.g. tabulating the study intervention characteristics and comparing against the planned groups for each synthesis (item #5)). | Methods  (Section 2.3)  Results  (Section 3.1) |
|  | 13b | Describe any methods required to prepare the data for presentation or synthesis, such as handling of missing summary statistics, or data conversions. | Methods  (Section 2.3) |
|  | 13c | Describe any methods used to tabulate or visually display results of individual studies and syntheses. | Methods  (Section 2.5) |
|  | 13d | Describe any methods used to synthesize results and provide a rationale for the choice(s). If meta-analysis was performed, describe the model(s), method(s) to identify the presence and extent of statistical heterogeneity, and software package(s) used. | Methods  (Section 2.5) |
|  | 13e | Describe any methods used to explore possible causes of heterogeneity among study results (e.g. subgroup analysis, meta-regression). | Methods  (Section 2.5) |
|  | 13f | Describe any sensitivity analyses conducted to assess robustness of the synthesized results. | Methods  (Section 2.5) |
| Reporting bias assessment | 14 | Describe any methods used to assess risk of bias due to missing results in a synthesis (arising from reporting biases). | Methods  (Section 2.5) |
| Certainty assessment | 15 | Describe any methods used to assess certainty (or confidence) in the body of evidence for an outcome. | Methods  (Section 2.6) |
| **RESULTS** | | |  |
| Study selection | 16a | Describe the results of the search and selection process, from the number of records identified in the search to the number of studies included in the review, ideally using a flow diagram. | Results  (Section 3.1)  Figure1  Supplementary Material  Table S1 |
|  | 16b | Cite studies that might appear to meet the inclusion criteria, but which were excluded, and explain why they were excluded. | Results  (Section 3.1)  Figure1 |
| Study characteristics | 17 | Cite each included study and present its characteristics. | Table1 Table2 |
| Risk of bias in studies | 18 | Present assessments of risk of bias for each included study. | Results  (Section 3.3)  Figure 2  Supplementary Material Table S2 |
| Results of individual studies | 19 | For all outcomes, present, for each study: (a) summary statistics for each group (where appropriate) and (b) an effect estimate and its precision (e.g. confidence/credible interval), ideally using structured tables or plots. | Results  (Section 3.4)  Figure3- Figure8 |
| Results of syntheses | 20a | For each synthesis, briefly summarise the characteristics and risk of bias among contributing studies. | Results  (Section 3.3) |
|  | 20b | Present results of all statistical syntheses conducted. If meta-analysis was done, present for each the summary estimate and its precision (e.g. confidence/credible interval) and measures of statistical heterogeneity. If comparing groups, describe the direction of the effect. | Table 3 |
|  | 20c | Present results of all investigations of possible causes of heterogeneity among study results. | Results  (Section 3.5) |
|  | 20d | Present results of all sensitivity analyses conducted to assess the robustness of the synthesized results. | Results  (Section 3.5) |
| Reporting biases | 21 | Present assessments of risk of bias due to missing results (arising from reporting biases) for each synthesis assessed. | Results  (Section 3.3) |
| Certainty of evidence | 22 | Present assessments of certainty (or confidence) in the body of evidence for each outcome assessed. | Results  (Section 3.6) |
| **DISCUSSION** | | |  |
| Discussion | 23a | Provide a general interpretation of the results in the context of other evidence. | Discussion |
|  | 23b | Discuss any limitations of the evidence included in the review. | Discussion |
|  | 23c | Discuss any limitations of the review processes used. | Discussion |
|  | 23d | Discuss implications of the results for practice, policy, and future research. | Discussion |
| **OTHER INFORMATION** | | |  |
| Registration and protocol | 24a | Provide registration information for the review, including register name and registration number, or state that the review was not registered. | Abstract  Methods  (Section 2.1) |
|  | 24b | Indicate where the review protocol can be accessed, or state that a protocol was not prepared. | - |
|  | 24c | Describe and explain any amendments to information provided at registration or in the protocol. | - |
| Support | 25 | Describe sources of financial or non-financial support for the review, and the role of the funders or sponsors in the review. | Founding |
| Competing interests | 26 | Declare any competing interests of review authors. | Competing interests |
| Availability of data, code and other materials | 27 | Report which of the following are publicly available and where they can be found: template data collection forms; data extracted from included studies; data used for all analyses; analytic code; any other materials used in the review. | Availability of data and materials |
